# Effects of topically administered 0.6% hyaluronic acid on the healing of labial frenectomy in conventional and 940-nm indium gallium arsenide phosphide (InGaAsP) diode laser techniques in pediatric patients: a randomized, placebo-controlled clinical study

**DOI:** 10.1007/s10103-024-03983-7

**Published:** 2024-01-27

**Authors:** Suat Serhan Altintepe Doğan, Nebi Cansın Karakan, Özgür Doğan

**Affiliations:** 1 Department of Periodontology, Faculty of Dentistry, Afyonkarahisar Health Sciences University, Güvenevler Mahallesi, İsmet İnönü St., No. 4, 03030 Afyonkarahisar, Turkey; 2 Deparment Of Pediatric Dentistry, Faculty of Dentistry, Afyonarahisar Health Sciences University, Afyonkarahisar, Turkey

**Keywords:** Hyaluronic acid gel, Frenectomy, Diode laser, Postoperative pain, Labial frenulum

## Abstract

This study aimed to investigate the effects of 0.6% hyaluronic acid (HA) gel on the healing process and postoperative pain levels after diode laser-assisted labial frenectomy in pediatric patients. Ninety-six pediatric patients (females, 50 and males, 46) aged 8–14 years were randomly divided into four groups as follows: (1) conventional frenectomy with 0.6% topically administered HA (CFH, *n *= 24); (2) conventional frenectomy with placebo gel (CFP, *n *= 24); (3) frenectomy performed by diode laser with 0.6% topically administered HA (DLH, *n *= 24); and (4) frenectomy performed by diode laser with placebo gel (DLP, *n *= 24). HA application was continued for 1 week thrice daily after the frenectomy. Visual analog scale forms were collected from patients 1 week after the operation. In addition, the plaque index, gingival index, periodontal probing depth, and keratinized tissue width and thickness were recorded. This process was repeated 1 and 3 months after the first visit. The DLH group revealed significant differences in the probing depth, bleeding on probing, keratinized gingiva width, and attached gingiva width according to dual comparisons of the initial, first, and third-month values (*p *= 0.010, *p *= 0.007, *p*<0.001, and *p *= 0.001, respectively). Significant differences were observed between the CFP and CFH groups according to the initial and initial third-month values with regard to the bleeding on probing (*p*=0.019 and *p *= 0.019, respectively). The attached gingival thickness revealed significant differences between the CFP and CFH groups for the initial and initial-third-month comparisons (*p *= 0.005 and *p *= 0.007, respectively). The mean values of the initial and initial-third-month differences were significantly higher in the CFH group than those in the CFP group. HA- and laser-assisted labial frenectomies revealed better outcomes in terms of the probing depth, attached gingiva width, keratinized gingiva width, healing process, and postoperative comfort.

## Introduction

The labial frenulum is an anatomical structure between the maxillary and mandibular central incisors and canine/premolar region. The frenulum is enclosed by a mucosal membrane and comprises fibrous, muscular, and fibromuscular tissues. This formation extends from the alveolar mucosa or attached gingiva to the underlying periosteum [1–3]. The labial frenulum attachments can be classified into four main categories according to the attachment level of the muscle fibers: mucosal, gingival, papillary, and papillary-penetrating (Table 1) [4].

**Table 1:** Morphological classification of the frenulum types [4]

Morphological classification of frenulum types	Frequency
Mucosal attachment (the frenulum fibers are attached up to the mucogingival junction)	42%
Gingival attachment (the frenulum fibers are inserted into the attached gingiva)	34%
Papillary attachment (the frenulum fibers extend into the interdental papilla)	20%
Papilla-penetrating attachment (the frenulum fibers cross the alveolar process and continue towards the palatine papilla)	4%

Papillary and papillary-penetrating types are identified as pathological when clinically diagnosed. Pathological labial frenulum attachments may lead to plaque accumulation, depending on the difficulty of tooth brushing due to muscle pulling, and may also cause diastema [5, 6]. Furthermore, papillary loss, gingival recession, midline diastema, and, in severe cases, periodontal pocket formation and tooth loss may occur due to the strong pulling of the muscle fibers over time [5]. If plaque control is poor, periodontal tissue loss may be more pronounced, especially in older individuals. High frenulum attachment combined with shallow sulcus may also create difficulties in tooth brushing [1, 7, 8]. In addition, high labial frenulum attachment in children with mixed dentition may cause more complicated problems, such as midline diastema, difficulty maintaining plaque control, and gingival recession [9]. Frenulectomy or frenectomy is recommended during the mixed dentition period, when the permanent teeth first erupt, to facilitate better oral hygiene, close midline diastemas spontaneously, and reduce the risk of caries [7, 8, 10]. It is used to surgically remove high frenulum attachments at the tissue level. Frenulectomy is characterized by a simple incision to release the frenulum attachment. However, frenectomy involves the removal of the entire frenulum, including attachments to the peritoneum and alveolar bone, in the cases having high frenulum [7]. The conventional technique of frenectomy involves using a scalpel [11]. Lasers are frequently used in frenectomy [3, 12]. In recent years, diode lasers have become more popular in frenectomies [2, 13]. In pediatric patients, diode lasers are commonly used for labial and lingual frenectomy because of the associated advantages such as reduced operation time, reduced need for infiltration anesthesia, hemostasis, and elimination of the need for suturing [2, 8]. The portability, ease of the setup process, and low price among the currently available lasers are other advantages of diode lasers [12].

In recent years, hyaluronic acid (HA) has been applied as an adjunct to oral soft tissues to enhance the healing process [4, 14, 15]. HA is a non-sulfated polysaccharide component of the glycosaminoglycans family. It is a high-molecular-weight polymer in various body fluids, such as synovial fluid, saliva, gingival crevicular fluid, and serum. HA is a significant component of the synovial joints, extracellular matrix of the skin, connective tissue, and various other tissues [16].

HA has bacteriostatic, fungistatic, anti-inflammatory, anti-edematous, and pro-angiogenetic effects on tissues. These properties suggest that HA may enhance wound healing in oral soft tissues, such as in the healing process following frenectomy operations [14, 16, 17]. Additionally, HA reduces plaque and sulcus bleeding indices in patients with gingivitis [18].

To the best of our knowledge, no previous studies assessed the advantages of the use of HA compared to control in laser and conventional frenectomy operations in pediatric patients. Accordingly, this study aimed at examining the effects of frenectomy and HA on clinical periodontal parameters. The primary purpose of this study was to evaluate the effects of HA on the healing process following different frenectomy methods using visual analog scale (VAS) scores and periodontal clinical parameters.

## Materials and methods

**Fig. 1:**
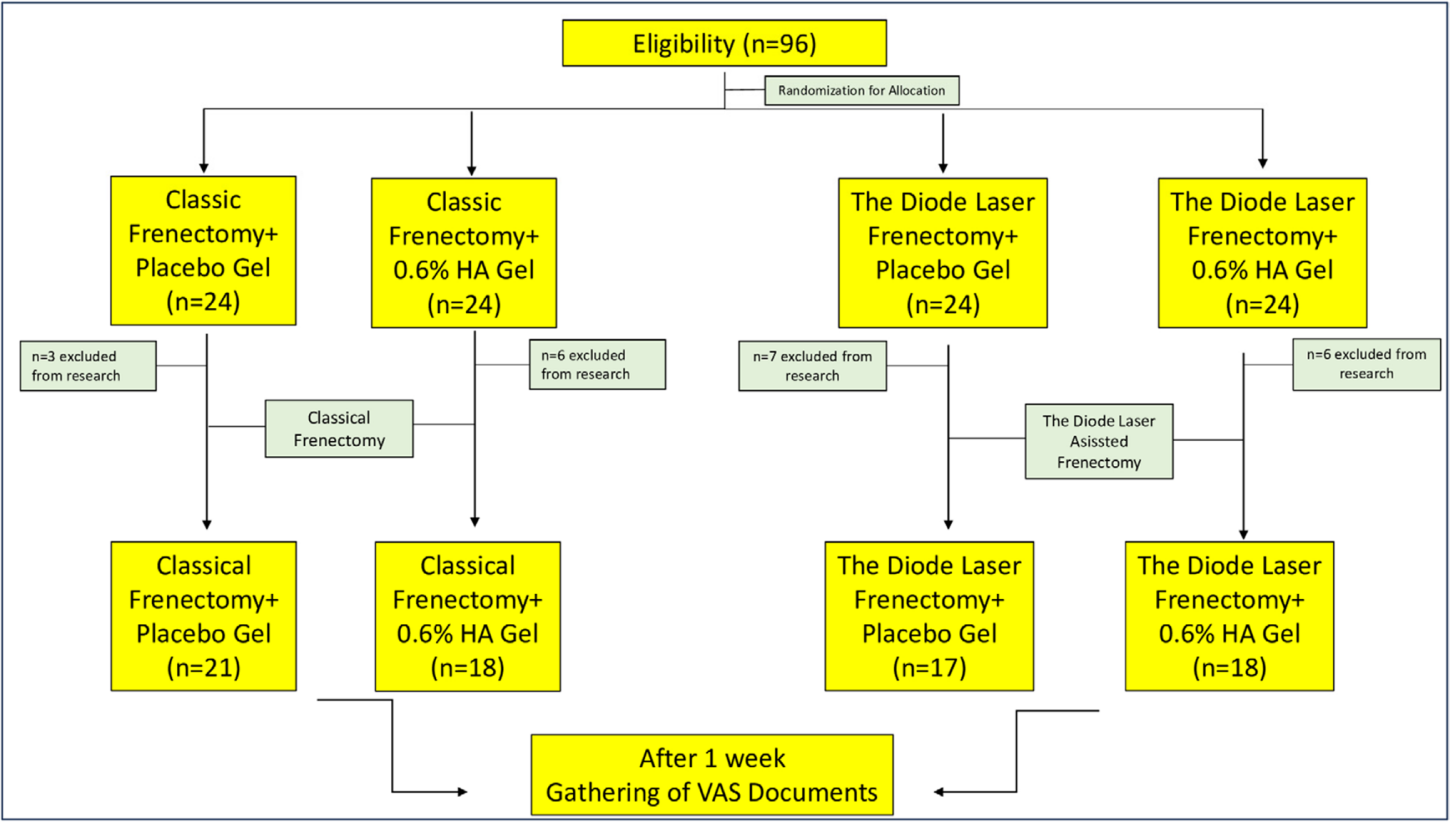
CONSORT diagram of the flow chart

This study was a prospective, examiner-blind, randomized, and controlled clinical trial. The local ethics committee approved the study protocol (Approval 2019/220). The clinical phases of the study were performed at the Department of the Periodontology Clinic between August 2019 and May 2021. The conducted research followed the ethical principles outlined in the Declaration of Helsinki. G-Power (version 3.1; Informer Tech Inc., Germany) was used to determine the number of research participants [19]. It was determined that each group must have a minimum of 16 individuals to achieve 95% power at a 5% significance level. To accommodate potential dropouts and non-compliance, 96 participants were recruited. The randomization sequence was generated using an online computer-based tool (www.random.org). The allocation of participants was independently conducted by individuals designated as OD, ensuring the concealment of allocation. Ninety-six pediatric patients (females, 50 and males, 46) aged 8–14 years were randomly divided into four groups: (1) conventional labial frenectomy with 0.6% topically administered HA (CFH, *n *= 24); (2) conventional labial frenectomy with placebo gel (CFP, *n *= 24); (3) labial frenectomy performed by diode laser with 0.6% topically administered HA (DLH, *n *= 24); and (4) labial frenectomy performed by diode laser with placebo gel (*n *= 24; Fig. 1).

Systemically healthy and non-medicated individuals (for at least 6 months) with gingival (*n *= 31; 32.29%), papillary (*n *= 39; 40.62%), and papillary penetrating (*n *= 26; 27.08%) labial frenulum attachments were included in the study. The parents signed a written informed consent form for their children’s participation in this study, and the children were given a brief description of the process. The study included frenectomy treatments performed by researchers SSAD and CA. The researchers performed frenectomy treatments on the children without knowing which group they belonged to (placebo or HA). Another researcher, other than the surgeon, performed control and postoperative measurements for each patient.

All the patients underwent dental treatment and initial periodontal therapy and followed the oral hygiene instructions. Before the labial frenectomy procedure, 10% lidocaine was topically applied to all the patients to reduce the pain before administering local anesthesia (Avixa İlaç San. Tic Ltd. Şti., Başakşehir, İstanbul, Turkey). Local infiltration anesthesia with 4% articaine, including epinephrine (epinephrine:articaine, 1:100,000), was applied to the vestibular oral mucosa and right and left regions of the frenulum (Ultracaine D-S Fort Ampul, Avixa İlaç San. Başakşehir, İstanbul, Turkey). The usage protocol for the diode laser was initiated after a waiting time of around 10 min.

**Fig. 2:**
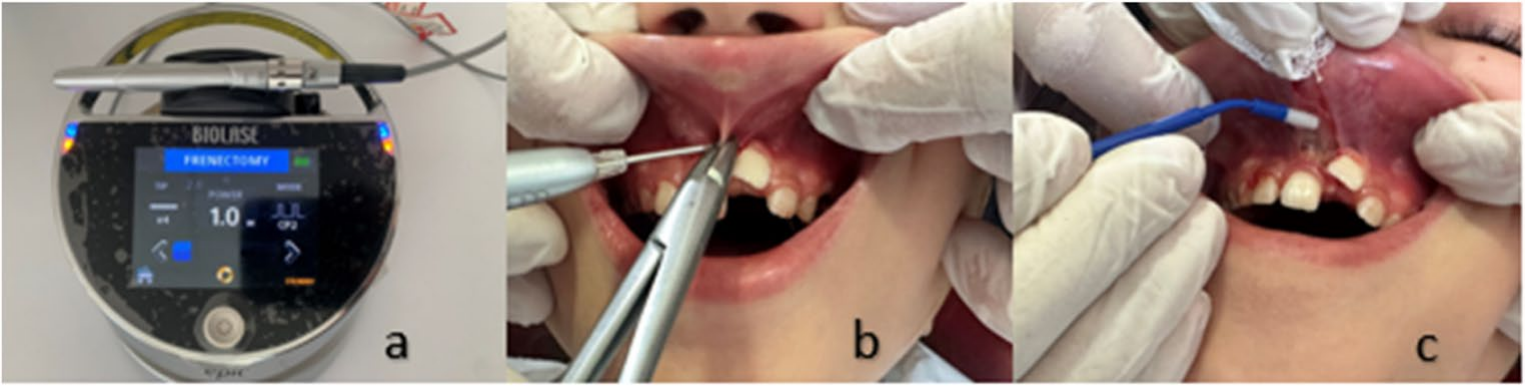
The frenectomy procedure stages utilizing the diode laser. **a** The Epic 10 diode laser. **b** Surgical procedure of the diode laser assisted-frenectomy. **c** The application of 0.6% HA gel as a demonstration for parents

The diode laser used in this study was the BIOLASE Epic10™ (BIOLASE INC., CA, USA). The laser interface was set to the “Frenectomy” mode. The features of the frenectomy mode are shown in Table 1. Frenectomy was performed using a 940-nm indium gallium arsenide phosphide (InGaAsP) semiconductor diode laser. The laser was operated in pulseCP2 wave mode at a power of 1.0 watt (W), and a 400-μm diameter optical fiber tip was used (Fig. 2). After the patient and operator wore protective glasses, the environmental safety measures were taken before carrying out the frenectomy procedure following the guidelines in Table 2.

**Table 2:** Parameters for the 940-nm diode laser

BIOLASE Epic 10™ diode soft tissue laser	
*Intrinsic parameters*	
Laser classification	IV
Medium	InGaAs semiconductor diode
Wavelength	940 ± 10 nm
Max power output	10 W
Power accuracy	± 20%
Power modes	Continuous, pulse modulation
Delivery system	The flexible optic fiber
Energy distribution	Quasi-flattop
Energy delivery	Non-initiated
Fiber tips diameter	200, 300, and 400 μm
Pulse duration	0.01–20 ms
Pulse interval	0.04–20 ms
Pulse repetition rate	Up to 20 kHz
Spot size (for surgical handpiece)	400 μm (maximum in contact mode)
Nominal ocular hazard distance	2.71 m
Maximum permissible exposure	30 W/m^2^
Beam divergence	7–22° per side angle
Aiming beam	Max. 1 mW, 625–670 nm, class 2
Standard fiber cable length	5 feet (1.5 m)
*Adjustable parameters*	
Frenectomy operating mode	Pulse mode
Used power	1.0 W
Irradiation mode	The activation occurs once the pedal is pressed and the targeted tissue is contacted.
Used optic fiber tip diameter	400 μm/7
Pulse duration	1 ms
Pulse interval	1 ms
Peak power	2.0 W
Average power	1.0 W
Beam divergence	8° per side
Speed of movement	2 mm/sec
*Calculated parameters*	
Total energy	60 J
Power density	⁓ 796 W/cm^2^
Average power density	100 W/cm^2^
Peak power density	200 W/cm^2^
Spot area at tissue	0.00126 cm^2^
Spot diameter at tissue	⁓ 0.04 cm
Tip area	0.005024 cm^2^
% on time	50%
Energy density	⁓ 1500 J/cm^2^

Using brushing movements, the laser was applied to the upper and lower parts of the frenulum near the hemostat. Moreover, the remaining muscular attachments of the periosteum were removed to eliminate the periosteal adhesion. The remaining ablated tissue was cleared using a moistened gauze with a sterile saline solution [2, 13]. The average time taken for the procedure with the diode laser was 60 s. After the surgical procedure, the participants were instructed on how to apply the HA gel. The patients in the 0.6% HA group were given seven blister disposable packages containing 0.6% HA (Aftamed Shield Gel, Aktident, Üsküdar, İstanbul, TURKEY). The participants in the HA group were instructed to apply HA Gel to the wound area thrice a day for 1 min after opening a new blister pack and refrain from eating or drinking for 10–15 min [4] (Fig. 2).

**Fig. 3:**
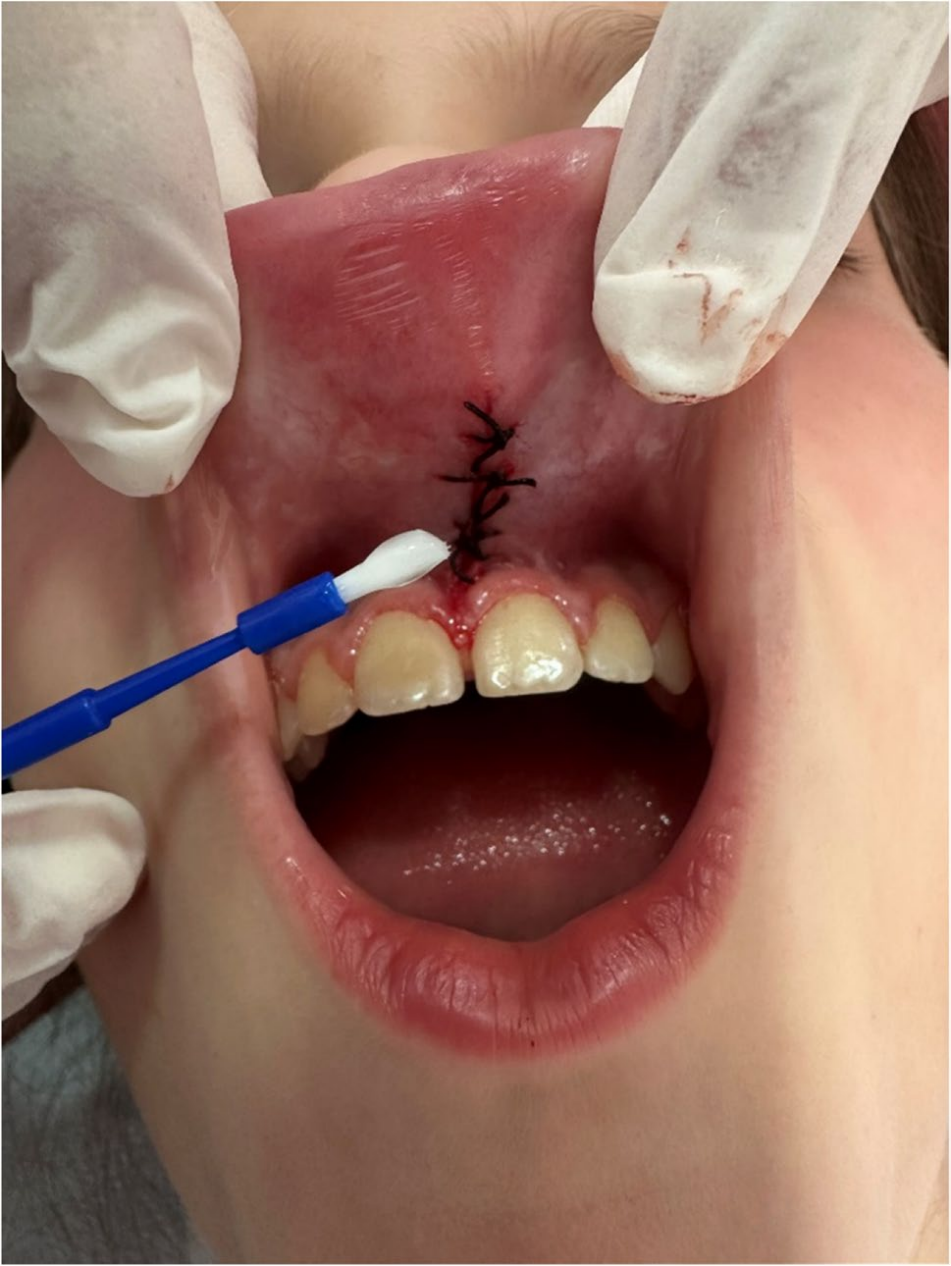
The application of 0.6% HA gel as a demonstration for parents after carrying out the classic frenectomy procedure

In conventional techniques, the upper lip is extended, and a straight hemostat is attached to the frenulum into the depth of the vestibular fold. Triangular-shaped incisions were made above and below the hemostat using a no. 15 scalpel (HM0240, Beybi, Ümraniye, İstanbul, Turkey) until the labial frenulum was released from the soft tissue. Muscle fiber dissection was performed on the submucosa of the lateral walls after excision of the frenulum with curved forceps to detach them from the periosteum. A 4/0 silk suture was used for primary wound closure (DOGSAN, Beşiktaş, İstanbul, Turkey). After surgical frenectomy was performed, the patients and their parents were advised to be cautious and avoid the exposure to mechanical trauma, flossing, and chewing movements. Gentle tooth brushing was permitted using a surgical toothbrush (Surgical Mega Soft, Curaprox, Kriens, Switzerland). The interrupted sutures were removed 1 week after surgery [1, 2, 13] (Fig. 3).

The Silness–Löe plaque index (PI), gingival index (GI), pocket depth (PD), bleeding on probing (BOP), keratinized gingival width (KGW), attached gingival width (AGW), and attached gingival thickness (AGT) were measured using a Williams periodontal sond (122-006, HuFriedy, Chic, IL, USA). They were recorded prior to the labial frenectomy operations and at 1 and 3 months after the operations [2, 13].

The VAS was used to evaluate the pain and discomfort levels of the patients, with scores between 0 and 10, where the score of “0” indicated no pain and discomfort and the score of “10” indicated severe pain and discomfort. The participants were requested to complete the questionnaire once daily for 1 week following the operation [2, 4, 13, 20].

Statistical analyses were performed using SPSS for Windows software package (version 20.0). For descriptive statistics, the mean ± standard deviation and median (minimum–maximum) were used for the quantitative variables, while the number of patients (percentage) was used for the qualitative variables. To determine whether there was a difference between the two categories of the qualitative and quantitative variables, Mann-Whitney *U* test was used as the normal distribution hypothesis was not provided. Repeated-measures analysis of variance (ANOVA) and two-way repeated-measures analysis of variance were used to examine the change in variables with repeated measures over time and between groups. A significance level of 0.05 was set for statistical analyses.

## Results

A total of 74 (77.08%) pediatric patients out of the 96 enrolled patients completed this study. Thus, 22 patients were excluded. One child did not correctly complete the VAS, another child did not use the placebo gel, and a third child missed the control appointment. Six children from the CFH group were excluded, where three children did not correctly use the 0.6% HA gel, two did not complete the VAS, and one missed the appointment. Seven children from the DFP group were excluded, where seven participants were excluded due to improper use of the placebo gel and two were excluded for incorrectly filling out the VAS. Six children were excluded from the DFH group, where three children were excluded due to improper use of HA, two did not appropriately fill out the VAS, and one missed the control appointment.

### Periodontal parameter assessments

In the CFP group, no significant differences were observed according to the dual comparisons of the measurements of the initial, first, and third months when all the variables were compared (*p*>0.05). There were significant differences in the CFH group for BOP between the values of the initial, first, and third months when dual comparisons were performed (*p*<0.001). According to the dual comparisons in the CFH group, the initial-first, initial-third, and first-third month comparisons showed significant differences (*p *= 0.004, *p *= 0.001, and *p *= 0.012, respectively; Table 3).

**Table 3:** Time comparisons between the CFP and CFH groups

Variables	Time	CFP			CFH		
		Mean ± SD	Median (min–max)	*p* value	Mean ± SD	Median (min–max)	*p* value
Plaque index	Initial	0.18 ± 0.51	0.00 (0.00 – 2.00)	0.277	0.16 ± 0.50	0.00 (0.00 – 2.00)	0.183
	First month	0.26 ± 0.45	0.00 (0.00 – 1.00)		0.19 ± 0.46	0.00 (0.00 – 2.00)	
	Third month	0.18 ± 0.39	0.00 (0.00 – 1.00)		0.13 ± 0.41	0.00 (0.00 – 2.00)	
Gingival index	Initial	0.13 ± 0.34	0.00 (0.00 – 1.00)	0.463	0.19 ± 0.46	0.00 (0.00 – 2.00)	0.156
	First month	0.16 ± 0.37	0.00 (0.00 – 1.00)		0.17 ± 0.45	0.00 (0.00 – 2.00)	
	Third month	0.08 ± 0.27	0.00 (0.00 – 1.00)		0.13 ± 0.41	0.00 (0.00 – 2.00)	
Probing depth	Initial	2.32 ± 0.62	2.00 (1.00 – 3.00)	0.422	2.19 ± 0.76	2.00 (0.00 – 4.00)	0.269
	First month	2.29 ± 0.57	2.00 (1.00 – 3.00)		2.26 ± 0.73	2.00 (0.00 – 4.00)	
	Third month	2.24 ± 0.68	2.00 (1.00 – 3.00)		2.20 ± 0.73	2.00 (0.00 – 4.00)	
Bleeding on probing	Initial	0.05 ± 0.23	0.00 (0.00 – 1.00)	0.658	0.53 ± 0.85	0.00 (0.00 – 3.00)	**<0.001**
	First month	0.05 ± 0.23	0.00 (0.00 – 1.00)		0.30 ± 0.52	0.00 (0.00 – 2.00)	
	Third month	0.03 ± 0.16	0.00 (0.00 – 1.00)		0.19 ± 0.46	0.00 (0.00 – 2.00)	
Keratinized gingiva width	Initial	5.29 ± 1.47	5.00 (1.00 – 7.00)	0.859	5.14 ± 1.14	5.00 (4.00 – 9.00)	0.509
	First month	5.53 ± 1.17	5.00 (4.00 – 8.00)		5.20 ± 0.99	5.00 (4.00 – 8.00)	
	Third month	5.47 ± 1.02	5.00 (4.00 – 7.00)		5.17 ± 0.92	5.00 (4.00 – 8.00)	
Attached gingiva width	Initial	4.61 ± 1.28	4.00 (3.00 – 8.00)	0.267	4.17 ± 1.15	4.00 (3.00 – 8.00)	0.502
	First month	4.53 ± 1.12	4.00 (3.00 – 7.00)		4.23 ± 1.00	4.00 (3.00 –7.00)	
	Third month	4.47 ± 0.96	4.00 (3.00 – 6.00)		4.23 ± 1.03	4.00 (3.00 – 8.00)	
Attached gingiva thickness	Initial	1.76 ± 0.53	2.00 (1.00 – 3.00)	0.086	1.96 ± 0.39	2.00 (1.00 – 3.00)	0.067
	First month	1.87 ± 0.46	2.00 (1.00 – 3.00)		1.87 ± 0.47	2.00 (1.00 – 3.00)	
	Third month	1.95 ± 0.50	2.00 (1.00 – 3.00)		1.93 ± 0.48	2.00 (1.00 – 3.00)	

Significance level, *p *< 0.05

The DLP group did not show significant differences in the variables when dual comparisons of the initial, first, and third months were performed (*p*>0.05). However, in the DLH group, significant differences were observed in the PD, BOP, KGW, and AGW according to the dual comparisons of the values of the initial, first, and third months (*p *= 0.010, *p *= 0.007, *p*<0.001, and *p *= 0.001, respectively). Dual comparisons of the probing depth values displayed significant differences between the first and third months (*p *= 0.028). The BOP value was significantly different between the initial and first months only (*p *= 0.011). The KGW values were significantly different between the initial-first and initial-third months (*p *= 0.034 and *p *= 0.030, respectively). Dual-time comparisons of the AGW values revealed significant differences between the initial and first months (*p *= 0.002; Table 4).

**Table 4:** Time comparisons for the DLP and DLH groups

Variables	Time	DLP			DLH		
		Mean ± SD	Median (min – max)	*p* value	Mean ± SD	Median (min – max)	*p* value
Plaque index	Initial	0.07 ± 0.26	0.00 (0.00 – 1.00)	0.434	0.26 ± 0.52	0.00 (0.00 – 2.00)	0.169
	First month	0.14 ± 0.52	0.00 (0.00 – 2.00)		0.41 ± 0.71	0.00 (0.00 – 2.00)	
	Third month	0.04 ± 0.19	0.00 (0.00 – 1.00)		0.37 ± 0.62	0.00 (0.00 – 2.00)	
Gingival index	Initial	0.07 ± 0.26	0.00 (0.00 – 1.00)	0.161	0.20 ± 0.41	0.00 (0.00 – 1.00)	0.155
	First month	0.00 ± 0.00	0.00 (0.00 – 0.00)		0.11 ± 0.32	0.00 (0.00 – 1.00)	
	Third month	0.00 ± 0.00	0.00 (0.00 – 0.00)		0.13 ± 0.34	0.00 (0.00 – 1.00)	
Probing depth	Initial	2.18 ± 0.48	2.00 (2.00 – 3.00)	0.437	2.15 ± 0.56	2.00 (1.00 – 3.00)	**0.010**
	First month	2.14 ± 0.45	2.00 (2.00 – 3.00)		2.11 ± 0.72	2.00 (1.00 – 3.00)	
	Third month	2.11 ± 0.50	2.00 (1.00 – 3.00)		2.33 ± 0.70	2.00 (1.00 – 5.00)	
Bleeding on probing	Initial	0.07 ± 0.26	0.00 (0.00 – 1.00)	0.161	0.19 ± 0.39	0.00 (0.00 – 1.00)	**0.007**
	First month	0.00 ± 0.00	0.00 (0.00 – 0.00)		0.04 ± 0.19	0.00 (0.00 – 1.00)	
	Third month	0.00 ± 0.00	0.00 (0.00 – 0.00)		0.07 ± 0.26	0.00 (0.00 – 1.00)	
Keratinized gingiva width	Initial	5.07 ± 0.77	5.00 (4.00 – 7.00)	0.183	4.94 ± 0.97	5.00 (3.00 – 7.00)	**<0.001**
	First month	5.18 ± 0.81	5.00 (4.00–7.00)		5.17 ± 0.94	5.00 (3.00 – 8.00)	
	Third month	5.14 ± 0.76	5.00 (4.00–6.00)		5.09 ± 0.87	5.00 (3.00 – 7.00)	
Attached gingiva width	Initial	4.07 ± 0.94	4.00 (2.00–6.00)	0.355	4.13 ± 1.36	4.00 (1.00 – 7.00)	**0.001**
	First month	4.14 ± 0.97	4.00 (2.00–6.00)		4.33 ± 1.30	4.00 (1.00 – 7.00)	
	Third month	4.11 ± 0.92	4.00 (2.00–5.00)		4.26 ± 1.24	4.00 (1.00 – 7.00)	
Attached gingiva thickness	Initial	1.61 ± 0.61	1.75 (1.00–3.00)	0.175	1.63 ± 0.48	2.00 (1.00 – 2.00)	0.086
	First month	1.57 ± 0.62	1.50 (1.00–3.00)		1.63 ± 0.49	2.00 (1.00 – 2.00)	
	Third month	1.68 ± 0.60	2.00 (1.00–3.00)		1.70 ± 0.54	2.00 (1.00 – 3.00)	

Significance level, *p *< 0.05

Significant differences were observed in the BOP values between the CFP and CFH groups according to the initial-first and initial-third months (*p *= 0.019 and *p *= 0.019, respectively). The CFP and CFH groups revealed differences in the mean values ( ± SD) of 0.00 ± 0.23 and 0.23 ± 0.57, respectively, of the initial-first month. Moreover, the CFP and CFH groups revealed differences in the mean values ( ± SD) of 0.03 ± 0.28 and 0.34 ± 0.78, respectively, of the initial-first month. The KGW values of initial-first month were significantly different between the CFP and CFH groups (*p *= 0.017). The CFP and CFH groups revealed differences in the mean values ( ± SD) of −0.03 ± 1.26 and −0.06 ± 0.41, respectively, of the initial-first month (Table 5).

**Table 5:** Comparisons of the time-dependent differences for the CFP and CFH groups

Variables	Time	CFP		CFH		
		Mean ± SD	Median (min – max)	Mean ± SD	Median (min – max)	*p value*
Plaque index	Initial-first month	−0.08 ± 0.43	0.00 (− 1.00 – 1.00)	− 0.03 ± 0.29	0.00 (− 1.00 – 1.00)	0.452
	Initial-third month	0.00 ± 0.33	0.00 (− 1.00 – 1.00)	0.03 ± 0.24	0.00 (− 1.00 – 1.00)	0.610
	First-third month	0.08 ± 0.27	0.00 (0.00 – 1.00)	0.06 ± 0.23	0.00 (0.00 – 1.00)	0.662
Gingival index	Initial-first month	−0.03 ± 0.49	0.00 (−1.00 – 1.00)	0.01 ± 0.27	0.00 (−1.00 – 1.00)	0.577
	Initial-third month	0.05 ± 0.46	0.00 (−1.00 – 1.00)	0.06 ± 0.23	0.00 (0.00 – 1.00)	1.000
	First-third month	0.08 ± 0.27	0.00 (0.00 – 1.00)	0.04 ± 0.27	0.00 (−1.00 – 1.00)	0.510
Probing depth	Initial-first month	0.03 ± 0.37	0.00 (−1.00 – 1.00)	−0.07 ± 0.46	0.00 (−2.00 – 1.00)	0.332
	Initial-third month	0.08 ± 0.36	0.00 (0.00–2.00)	−0.01 ± 0.32	0.00 (−1.00 – 1.00)	0.246
	First-third month	0.05 ± 0.40	0.00 (−1.00 – 1.00)	0.06 ± 0.38	0.00 (−1.00 – 2.00)	0.891
Bleeding on probing	Initial-first month	0.00 ± 0.23	0.00 (−1.00 – 1.00)	0.23 ± 0.57	0.00 (0.00 – 2.00)	**0.019**
	Initial-third month	0.03 ± 0.28	0.00 (−1.00 – 1.00)	0.34 ± 0.78	0.00 (0.00 – 3.00)	**0.019**
	First-third month	0.03 ± 0.16	0.00 (0.00 – 1.00)	0.11 ± 0.32	0.00 (0.00 – 1.00)	0.116
Keratinized gingiva width	Initial-first month	−0.03 ± 1.26	0.00 (− 7.00 – 2.00)	−0.06 ± 0.41	0.00 (−1.00 – 2.00)	**0.017**
	Initial-third month	0.03 ± 1.21	0.00 (−6.00 – 3.00)	−0.03 ± 0.51	0.00 (−1.00 – 2.00)	0.106
	First-third month	0.05 ± 0.40	0.00 (−1.00 – 1.00)	0.03 ± 0.34	0.00 (−1.00 – 1.00)	0.732
Attached gingiva width	Initial-first month	0.08 ± 0.41	0.00 (−1.00 – 1.00)	−0.06 ± 0.41	0.00 (−1.00 – 2.00)	**0.031**
	Initial-third month	0.13 ± 0.65	0.00 (−1.00 – 2.00)	−0.04 ± 0.52	0.00 (−1.00 – 2.00)	0.122
	First-third month	0.05 ± 0.40	0.00 (−1.00 – 1.00)	0.01 ± 0.36	0.00 (−1.00 – 1.00)	0.607
Attached gingiva thickness	Initial-first month	−0.11 ± 0.31	0.00 (−1.00 – 0.00)	0.09 ± 0.33	0.00 (−1.00 – 1.00)	**0.005**
	Initial-third month	−0.18 ± 0.46	0.00 (−1.00 – 1.00)	0.03 ± 0.34	0.00 (−1.00 – 1.00)	**0.007**
	First-third month	−0.08 ± 0.36	0.00 (−1.00 – 1.00)	−0.06 ± 0.23	0.00 (−1.00 – 0.00)	0.677

Significance level, *p *< 0.05

The AGW was significantly different between the CFP and CFH groups in the initial-first month (*p *= 0.031). The mean value of the initial-first month difference in the CFP group was significantly higher than that in the CFH group (Table 5).

The AGT values revealed significant differences between the CFP and CFH groups for the initial-first and initial-third month comparisons (*p *= 0.005 and *p *= 0.007, respectively). The differences in the mean values of the initial-first and initial-third months were significantly higher in the CFH group than those in the CFP group (Table 5).

Significant differences between the DLP and DLH groups were observed only for the PD according to the comparisons of the initial-third and first-third months (*p *= 0.029 and *p *= 0.029, respectively). The differences in the mean values (±SD) of the initial-third month in the DLP and DLH groups were 0.07 ± 0.38 and −0.19 ± 0.59, respectively. The difference in the mean value of the first-third month was significantly higher in the DLP group than that in the DLH group (Table 6).

**Table 6:** Comparisons between the time-dependent differences in the DLP and DLH groups

Variables	Time	DLP		DLH		
		Mean ± SD	Median (min–max)	Mean ± SD	Median (min–max)	*p* value
Plaque index	Initial-first month	− 0.07 ± 0.60	0.00 ( − 2.00 – 1.00)	− 0.15 ± 0.66	0.00 ( − 2.00 – 1.00)	0.442
	Initial-third month	0.04 ± 0.19	0.00 (0.00 – 1.00)	− 0.11 ± 0.60	0.00 ( − 2.00 – 1.00)	0.254
	First month-third month	0.11 ± 0.57	0.00 ( − 1.00 – 2.00)	0.04 ± 0.51	0.00 ( − 1.00 – 2.00)	0.811
Gingival index	Initial- first month	0.07 ± 0.26	0.00 (0.00 – 1.00)	0.09 ± 0.29	0.00 (0.00 – 1.00)	0.747
	Initial-third month	0.07 ± 0.26	0.00 (0.00 – 1.00)	0.07 ± 0.43	0.00 ( − 1.00 – 1.00)	0.936
	First month-third month	0.00 ± 0.00	0.00 (0.00 – 0.00)	− 0.02 ± 0.36	0.00 ( − 1.00 – 1.00)	0.778
Probing depth	Initial-first month	0.04 ± 0.19	0.00 (0.00 – 1.00)	0.04 ± 0.43	0.00 ( − 1.00 – 1.00)	0.960
	Initial-third month	0.07 ± 0.38	0.00 ( − 1.00 – 1.00)	− 0.19 ± 0.59	0.00 ( − 3.00 – 1.00)	**0.029**
	First month-third month	0.04 ± 0.33	0.00 ( − 1.00 – 1.00)	− 0.22 ± 0.60	0.00 ( − 3.00 – 0.00)	**0.029**
Bleeding on probing	Initial-first month	0.07 ± 0.26	0.00 (0.00 – 1.00)	0.15 ± 0.36	0.00 (0.00 – 1.00)	0.317
	Initial-third month	0.07 ± 0.26	0.00 (0.00 – 1.00)	0.11 ± 0.37	0.00 ( − 1.00 – 1.00)	0.593
	First-third month	0.00 ± 0.00	0.00 (0.00 – 0.00)	− 0.04 ± 0.19	0.00 ( − 1.00 – 0.00)	0.306
Keratinized gingiva width	Initial-first month	− 0.11 ± 0.28	0.00 ( − 1.00 – 0.00)	− 0.22 ± 0.42	0.00 ( − 1.00 – 0.00)	0.306
	Initial-third month	− 0.07 ± 0.38	0.00 ( − 1.00 – 1.00)	− 0.15 ± 0.41	0.00 ( − 1.00 – 1.00)	0.410
	First-third month	0.04 ± 0.23	0.00 ( − 0.50 – 1.00)	0.07 ± 0.33	0.00 ( − 1.00 – 1.00)	0.595
Attached gingiva width	Initial-first month	− 0.07 ± 0.26	0.00 ( − 1.00 – 0.00)	− 0.20 ± 0.41	0.00 ( − 1.00 – 0.00)	0.122
	Initial-third month	− 0.04 ± 0.33	0.00 ( − 1.00 – 1.00)	− 0.13 ± 0.39	0.00 ( − 1.00 – 1.00)	0.278
	First-third month	0.04 ± 0.19	0.00 (0.00 – 1.00)	0.07 ± 0.26	0.00 (0.00 – 1.00)	0.494
Attached gingiva thickness	Initial-first month	0.04 ± 0.33	0.00 ( − 1.00 – 1.00)	0.00 ± 0.26	0.00 ( − 1.00 – 1.00)	0.470
	Initial-third month	− 0.07 ± 0.26	0.00 ( − 1.00 – 0.00)	− 0.07 ± 0.31	0.00 ( − 1.00 – 1.00)	0.787
	First-third month	− 0.11 ± 0.31	0.00 ( − 1.00 – 0.00)	− 0.07 ± 0.26	0.00 ( − 1.00 –0.00)	0.614

Significance level, *p *< 0.05

Only the KGW parameter showed a significant difference between the CFP and DLP groups in the first month (*p *= 0.016). The initial-first month difference for KGW was −0.03±1.26 in the CFP group and −0.11±0.28 in the DLP group (Table 7).

**Table 7:** Comparisons of the time-dependent differences in the CFP and DLP groups

Variables	Time	CFP		DLP		
		Mean±SD	Median (min–max)	Mean±SD	Median (min–max)	*p* value
Plaque index	Initial-first month	−0.08±0.43	0.00 (−1.00–1.00)	−0.07±0.60	0.00 (−2.00–1.00)	0.496
	Initial-third month	0.00±0.33	0.00 (−1.00–1.00)	0.04±0.19	0.00 (0.00–1.00)	0.611
	First-third month	0.08±0.27	0.00 (0.00–1.00)	0.11±0.57	0.00 (−1.00–2.00)	0.630
Gingival index	Initial-first month	−0.03±0.49	0.00 (−1.00–1.00)	0.07±0.26	0.00 (0.00–1.00)	0.347
	Initial-third month	0.05±0.46	0.00 (−1.00–1.00)	0.07±0.26	0.00 (0.00–1.00)	0.884
	First-third month	0.08±0.27	0.00 (0.00–1.00)	0.00±0.00	0.00 (0.00–0.00)	0.131
Probing depth	Initial-first month	0.03±0.37	0.00 (−1.00–1.00)	0.04±0.19	0.00 (0.00–1.00)	0.917
	Initial-third month	0.08±0.36	0.00 (0.00–2.00)	0.07±0.38	0.00 (−1.00–1.00)	0.805
	First-third month	0.05±0.40	0.00 (−1.00–1.00)	0.04±0.33	0.00 (−1.00–1.00)	0.845
Bleeding on probing	Initial-first month	0.00±0.23	0.00 (−1.00–1.00)	0.07±0.26	0.00 (0.00–1.00)	0.246
	Initial-third month	0.03±0.28	0.00 (−1.00–1.00)	0.07±0.26	0.00 (0.00–1.00)	0.515
	First-third month	0.03±0.16	0.00 (0.00–1.00)	0.00±0.00	0.00 (0.00–0.00)	0.391
Keratinized gingiva width	Initial-first month	−0.03±1.26	0.00 (−7.00–2.00)	−0.11±0.28	0.00 (−1.00–0.00)	**0.016**
	Initial-third month	0.03±1.21	0.00 (−6.00–3.00)	−0.07±0.38	0.00 (−1.00–1.00)	0.110
	First-third month	0.05±0.40	0.00 (−1.00–1.00)	0.04±0.23	0.00 (−0.50–1.00)	0.828
Attached gingiva width	Initial-first month	0.08±0.41	0.00 (−1.00–1.00)	−0.07±0.26	0.00 (−1.00–0.00)	0.067
	Initial-third month	0.13±0.65	0.00 (−1.00–2.00)	−0.04±0.33	0.00 (−1.00–1.00)	0.222
	First-third month	0.05±0.40	0.00 (−1.00–1.00)	0.04±0.19	0.00 (0.00–1.00)	0.808
Attached gingiva thickness	Initial-first month	−0.11±0.31	0.00 (−1.00–0.00)	0.04±0.33	0.00 (−1.00–1.00)	0.085
	Initial-third month	−0.18±0.46	0.00 (−1.00–1.00)	−0.07±0.26	0.00 (−1.00–0.00)	0.221
	First-third month	−0.08±0.36	0.00 (−1.00–1.00)	−0.11±0.31	0.00 (−1.00–0.00)	0.757

Significance level, *p *< 0.05

The difference in PD between the CFH and DLH groups was significantly higher in the initial-third month than that in the CFH group (*p *= 0.002). There was a difference in the probing depth between the CFH and DLH groups, with average values of 0.06±0.38 and −0.22±0.60, respectively, during the first-third month. The difference in BOP between the CFH and DLH groups was significant during the first 3 months (*p *= 0.003). While the CFH group had a difference in the average value for BOP in the first-third month of 0.11±0.32, the mean value for the DLH group was −0.04±0.19. The CFH and DLH groups showed a significant difference in KGW in the initial-first month (*p *= 0.034). The KGW value showed a difference of −0.06±0.41 in the CFH group and −0.22±0.42 in the DLH group during the initial-first month (Table 8).

**Table 8:** Comparisons of the time-dependent differences in the CFH and DLH groups

Variables	Time	CFH		DLH		
		Mean ± SD	Median (min–max)	Mean±SD	Median (min–max)	*p* value
Plaque index	Initial-first month	−0.03 ± 0.29	0.00 (−1.00– 1.00)	−0.15 ± 0.66	0.00 (−2.00 – 1.00)	0.327
	Initial-third month	0.03 ± 0.24	0.00 (−1.00 – 1.00)	−0.11 ± 0.60	0.00 (−2.00 – 1.00)	0.130
	First-third month	0.06 ± 0.23	0.00 (0.00 – 1.00)	0.04 ± 0.51	0.00 (−1.00 – 2.00)	0.587
Gingival index	Initial-first month	0.01 ± 0.27	0.00 (−1.00 – 1.00)	0.09 ± 0.29	0.00 (0.00 – 1.00)	0.127
	Initial-third month	0.06 ± 0.23	0.00 (0.00 – 1.00)	0.07 ± 0.43	0.00 (−1.00 – 1.00)	0.727
	First-third month	0.04 ± 0.27	0.00 (−1.00 – 1.00)	−0.02 ± 0.36	0.00 (−1.00 – 1.00)	0.282
Probing depth	Initial-first month	−0.07 ± 0.46	0.00 (−2.00 – 1.00)	0.04 ± 0.43	0.00 (−1.00 – 1.00)	0.250
	Initial-third month	−0.01 ± 0.32	0.00 (−1.00 – 1.00)	−0.19 ± 0.59	0.00 (−3.00 – 1.00)	0.066
	First-third month	0.06 ± 0.38	0.00 (−1.00 – 2.00)	−0.22 ± 0.60	0.00 (−3.00 – 0.00)	**0.002**
Bleeding on probing	Initial-first month	0.23 ± 0.57	0.00 (0.00 – 2.00)	0.15 ± 0.36	0.00 (0.00 – 1.00)	0.766
	Initial-third month	0.34 ± 0.78	0.00 (0.00 – 3.00)	0.11 ± 0.37	0.00 (−1.00 – 1.00)	0.160
	First-third month	0.11 ± 0.32	0.00 (0.00 – 1.00)	−0.04 ± 0.19	0.00 (−1.00 – 0.00)	**0.003**
Keratinized gingiva width	Initial-first month	−0.06 ± 0.41	0.00 (−1.00 – 2.00)	−0.22 ± 0.42	0.00 (−1.00 – 0.00)	**0.034**
	Initial-third month	−0.03 ± 0.51	0.00 (−1.00 – 2.00)	−0.15 ± 0.41	0.00 (−1.00 – 1.00)	0.202
	First-third month	0.03 ± 0.34	0.00 (−1.00 – 1.00)	0.07 ± 0.33	0.00 (−1.00 – 1.00)	0.457
Attached gingiva width	Initial-first month	−0.06 ± 0.41	0.00 (−1.00 – 2.00)	−0.20 ± 0.41	0.00 (−1.00 – 0.00)	0.059
	Initial-third month	−0.04 ± 0.52	0.00 (−1.00 – 2.00)	−0.13 ± 0.39	0.00 (−1.00 – 1.00)	0.393
	First-third month	0.01 ± 0.36	0.00 (−1.00 – 1.00)	0.07 ± 0.26	0.00 (0.00 – 1.00)	0.319
Attached gingiva thickness	Initial-first month	0.09 ± 0.33	0.00 (−1.00 – 1.00)	0.00 ± 0.26	0.00 (−1.00 – 1.00)	0.077
	Initial-third month	0.03 ± 0.34	0.00 (−1.00 – 1.00)	−0.07 ± 0.31	0.00 (−1.00 – 1.00)	0.059
	First-third month	−0.06 ± 0.23	0.00 (−1.00 – 0.00)	−0.07 ± 0.26	0.00 (−1.00 – 0.00)	0.705

Significance level, *p *< 0.05

Although time comparisons did not show a significant difference between the CFH and DLH groups, the CFH group exhibited worse PI, GI, PD, and BOP values. The treatment did not cause any significant changes in the KGW, ATW, or AGT values. Using HA reduces periodontal inflammation in diode laser-assisted frenectomy procedures, especially within the first month, where the patients showed increased attention to plaque cleaning, especially in the DLH group, when evaluating the time-dependent differences. The use of diode laser and HA gel had a positive impact on PI, GI, PD, BOP, KGW, and AGW values, particularly in the first month.

### VAS results

**Fig. 4:**
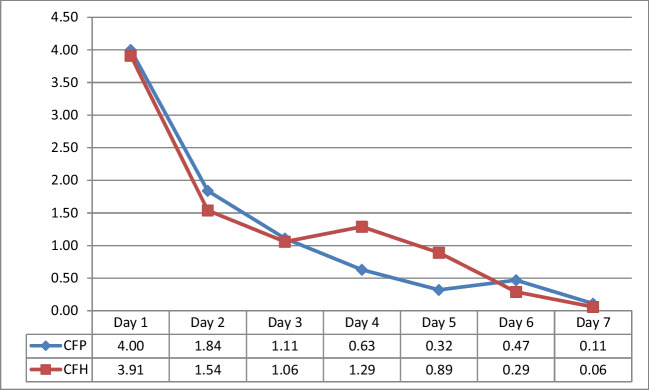
Alteration in the VAS in the CFP and CFH groups

VAS data showed significant differences in the CFP group between days 1–7 (*p*<0.001) and 2–7 (*p*<0.001). Additionally, there was a significant difference between days 2–4 (*p *= 0.007), 2–5 (*p*<0.001), and 2–6 (*p *= 0.001; Fig. 4).

Significant differences were observed in the CFH group between various pairs of days, including the 1st–7th (*p*<0.001), 2nd–7th (*p*<0.001), and 4th–7th days (*p*<0.001; Fig. 4).

**Fig. 5:**
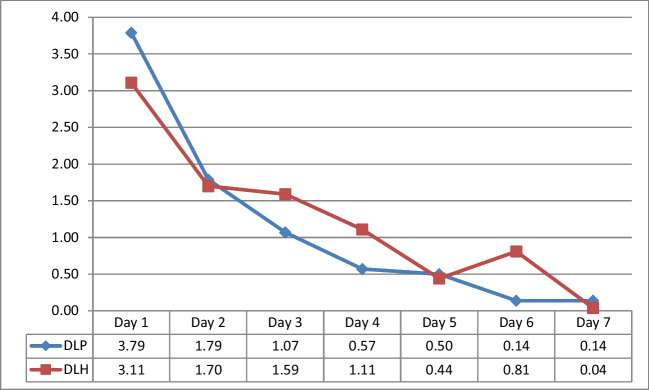
Alterations in the VAS in the DLP and DLH groups

There were significant differences in the DLP group between several days (1st–2nd, 1st–3rd, 1st–4th, 1st–5th, 1st–6th, 1st–7th, 2nd–4th, 2nd–5th, 2nd–6th, 2nd–7th, 3rd–6th, and 3rd–7th; all *p*<0.005; Fig. 5) The DLH group showed significant differences between days 1–7, with the most significant differences observed between days 1–5 and 3–7 (all *p*<0.005; Fig. 5).

The study analyzed the changes in the VAS measurements between the paired groups over time. There was no significant change between the CFP and CFH groups (*p *= 0.183). At each time, the VAS values of the CFH method were on average 0.08 units higher than those of the CFP method. There was a significant difference between the two groups only in the VAS value on day 4 (*p *= 0.041). No significant difference was observed between the DLP and DLH groups at all the time points (*p = *0.088). The VAS values of the DLH method were on average 0.12 units higher than those of the DLP method at all the time points. There was no significant difference in the VAS values between the two groups at all the time points (*p*>0.05). No significant differences were observed between the CFP and DLP groups at all the time points (*p *= 0.231). The VAS values were consistently 0.07 units higher with the CFP method than those with the DLP method. There was no significant difference in the VAS values between the two groups at all the time points (*p*>0.05). There was a significant difference in the rate of change over time between the CFH and DLH groups (*p *= 0.009). The two groups showed different trends in the increase or decrease of VAS over time. The VAS values of the CFH method were consistently 0.03 units higher than those of the DLH method. There was no significant difference in the VAS values between the two groups at all the time points (*p*>0.05; Figs. 1 and 2).

It is understood that using HA gel can reduce pain levels by suppressing inflammation, particularly on the first day. However, it was determined that inflammation was suppressed from the 4th day onwards, and the pain level was less. However, the same situation is not valid for days 2 and 3.

## Discussion

The labial frenulum abnormalities may cause midline diastema, gingival recession, and speech problems depending on the size and location of the frenulum [9]. Excision of the labial frenulum attachment is occasionally required in patients at the mixed-dentition stage to eliminate the incidence of these abnormalities [10]. The primary purpose of frenectomy is to minimize the tension of the soft tissue attachment to the underlying alveolar bone by excising the collagen fibers. Scalpels are commonly used to remove soft tissues using conventional techniques [13]. However, conventional techniques can cause postoperative pain and discomfort [7]. Laser-assisted frenectomy is a suitable alternative method for reducing the postoperative discomfort experienced by the patients [4].

Furthermore, laser-assisted surgery eliminates bleeding and the need for suturing [1, 6]. Previous studies reported that laser-assisted frenectomy yielded better outcomes than those of the scalpel technique for postoperative pain and functional complications [1, 13, 21]. In contrast, no significant differences were observed between the Nd-YAG laser and conventional frenectomy groups regarding postoperative pain or oral functions [3, 21]. The VAS, which is commonly used to evaluate the levels of pain and discomfort, is a reliable method that can be easily performed by patients and researchers [1, 21]. Recently, diode lasers have exhibited better outcomes than CO_2_ lasers for wound healing, clinical attachment level, gingival recession improvement, and postoperative comfort in labial frenectomy [12]. The diode laser has a superior advantage in terms of not interacting with hard tissues [22].

Furthermore, diode lasers reduce the need for infiltration anesthesia and, consequently, reduce anxiety in pediatric patients [8]. In this study, the pain levels were reduced in all the groups over time, but no significant differences were observed. As predicted, the most evident differences were observed after the first day.

The HA concentration in the commercially available products containing high molecular weight HA is in the range of 0.2–0.8% [16, 18]. The concentration, treatment protocol, and frequency may have affected the different clinical results reported in the previous studies [23]. Significant pain reduction was observed in a study in which 0.2% and 0.8% HA were used to reduce pain and burning sensation and accelerate palatal epithelial wound healing following a free gingival graft operation [15]. Subgingival administration of 0.2 mL 0.8% HA gel significantly reduced gingival bleeding [24] and gingival crevicular fluid flow rate [25] compared to the control groups in the patients with chronic periodontitis. Similarly, applying 0.2-mL 0.8% HA gel for 1 week after scaling and root planning significantly reduced BOP, PI, pocket PD, clinical attachment level, and colony-forming units [26]. The topical administration of 0.8% HA twice daily for 3 weeks significantly enhanced the plaque index and bleeding when probing the gingival crevicular fluid flow rate parameters in patients with gingivitis [27]. Local application of 0.8% HA twice daily for 4 weeks revealed similar results for the gingival index compared to those of the placebo gel group [26]. In a similar study, topical application of 0.2% HA as an adjunct to initial periodontal therapy in patients with chronic periodontitis revealed significantly better results when compared with those of the control group [28]. In a study investigating the effects of 0.2% HA on recurrent aphthous ulcers and oral ulcers that occur in Behçet disease, HA had positive effects on the number of ulcerative lesions, healing period, VAS, pain level, maximum area of ulcers, and inflammatory symptoms [29]. In the present study, 0.6% HA was applied following labial frenectomy operations. This dosage was selected based on similar previous studies. Moreover, the effect of 0.6% HA gel on the periodontal parameters after diode-laser-assisted frenectomy has not been previously reported in the literature.

HA may accelerate wound healing through blood clot stabilization, decreased inflammatory responses, neovascularization, angiogenesis, increased fibroblast migration, and wound closure [16, 18]. A meta-analysis reported that the laser-assisted labial frenectomy decreased the need for the administration of postoperative analgesics; however, no significant difference was observed between the laser-assisted frenectomy and conventional technique. On the other hand, the operation time, pain level, and discomfort during speech and chewing revealed better results with the laser-assisted frenectomy than those with the conventional technique [11]. Only one study reported that a better and faster healing process was observed in the patients who underwent labial frenectomy using the conventional scalpel technique [30]. This outcome may be explained by the carbonization effect of the laser, which retarded the healing process in the early stages [4]. In addition, primary closure using the conventional techniques may provide better outcomes during the early healing period [30].

Pain is a subjective condition that is difficult to evaluate objectively because the threshold level shows interindividual variations. Self-report measures are considered the most accurate and reliable method for assessing pain in children [20]. There are different methods of verbal self-report measures, including structured interviews, questionnaires, self-rating scales, and pain adjective descriptors. These methods can help individuals describe and communicate their pain experiences more accurately [31]. A child’s ability to self-report pain effects emerges later than pain intensity. These measures are typically introduced at the age of 5 years or older [32]. The VAS, which is frequently used to determine the pain level, is an easy and practical method that reveals specific outcomes depending on the clear explanation of the study and pain level evaluation process for the patient. Children are capable of assessing pain intensity at an approximate age of 8 years [33]. Children aged 8–14 years completed the VAS under parental supervision in the present study [34].

Only one previous study compared two different frenectomy techniques that evaluate the KGW, AGW, and AGT [13]. The HA- and laser-assisted frenectomy interactions were evaluated in adults without using conventional techniques in one study only [4]. In the present study, the PI, GI, BOP, and probing depth were included, and the VAS values were assessed. Furthermore, to our knowledge, no previous studies evaluated the HA effect on diode laser-assisted and conventional frenectomy techniques using these parameters in the healing process in pediatric patients.

BOP showed a significant time-dependent reduction in the CFH and DLH groups, which can be explained by the effects of HA on the inflammation phase and wound healing process rather than the enhancement of oral hygiene habits [18]. Enhancement of the probing depth, AGW, and KGW values was prominently observed in the groups that underwent HA and laser application. HA- and laser-assisted labial frenectomy displayed better tendencies than the conventional techniques regarding the postoperative patient comfort and periodontal soft tissue healing process.

This study has some limitations. First, there was a limited number of enrolled patients. The researchers faced a notable challenge while conducting studies with a larger group of participants. Second, keeping children calm and relaxed during dental procedures was challenging, especially when using laser. This could be attributed to the requirement of administering sedation to the patients. Third, the behavioral disorder caused by wearing protective equipment, including glasses, was another contributing factor. The behavioral management technique was applied to all patients (by OD). Fourth, some patients tend to avoid using HA gel regularly as communicated by some parents to the researchers. These patients were excluded from the study. Fifth, the study was concluded with a follow-up duration of 3 months. After the frenectomy procedure, many patients missed their appointments and were excluded from the study. Due to uncertainty about the COVID-19 pandemic duration, the follow-up period was short. Studies with longer follow-up durations focus on the KGW parameter; only one study by Uraz *et al.* reported recurrence in a few patients following diode laser-assisted frenectomy for 3 months of follow-up [13]. However, Pie-Sanchez *et al.* [35], Sezgin *et al*. [6], and Öztürk Özener *et al*. [2] stated that there was no recurrence in the KGW parameter after a follow-up period of 12 months. It is worth noting that all the studies were solely conducted on adult patients. If the follow-up period was longer, it may be easier to understand parameter changes in pediatric patients as they grow and develop. Finally, our results were obtained from pediatric patients. Although the VAS forms were filled out under parental supervision, the effectiveness of this measure depended on the accuracy of reporting by the family and children at home. However, this limitation can be eliminated by increasing the number of participants in future studies.

## Conclusion

Diode laser-assisted labial frenectomy can be performed in pediatric patients to reduce the postoperative pain levels. Using 0.6% HA as an adjunct to diode laser-assisted frenectomy can have positive alleviating effects on the postoperative pain levels, soft tissue healing, and periodontal parameters. Evaluating the long-term development of patients under the supervision of an orthodontist will enhance the evaluation of changes in the value of the KGW parameter in future studies.
